# Covalent Coupling of Nanoparticles with Low-Density Functional Ligands to Surfaces via Click Chemistry

**DOI:** 10.3390/ijms14023705

**Published:** 2013-02-07

**Authors:** Ina Rianasari, Michel P. de Jong, Jurriaan Huskens, Wilfred G. van der Wiel

**Affiliations:** + Institute for Nanotechnology, University of Twente, P.O. Box 217, 7500 AE Enschede, The Netherlands; E-Mails: i.rianasari@utwente.nl (I.R.); m.p.dejong@utwente.nl (M.P.J.); + Institute for Nanotechnology, University of Twente, P.O. Box 217, 7500 AE Enschede, The Netherlands; 1 NanoElectronics (NE) Group, MESA; 2 Molecular NanoFabrication Group, MESA

**Keywords:** gold nanoparticles, ligand coverage, click chemistry, microcontact printing, monolayers

## Abstract

We demonstrate the application of the 1,3-dipolar cycloaddition (“click” reaction) to couple gold nanoparticles (Au NPs) functionalized with low densities of functional ligands. The ligand coverage on the citrate-stabilized Au NPs was adjusted by the ligand:Au surface atom ratio, while maintaining the colloidal stability of the Au NPs in aqueous solution. A procedure was developed to determine the driving forces governing the selectivity and reactivity of citrate-stabilized and ligand-functionalized Au NPs on patterned self-assembled monolayers. We observed selective and remarkably stable chemical bonding of the Au NPs to the complimentarily functionalized substrate areas, even when estimating that only 1–2 chemical bonds are formed between the particles and the substrate.

## 1. Introduction

Limitations in lithographic technology have shifted the fabrication of nanoelectronic structures from purely top-down routes to the bottom-up assembly of hybrid, functional nanomaterials. Colloidal gold nanoparticles (Au NPs) have been recognized as attractive building blocks for nanoscale devices, mainly due to their unique optoelectronic [1,2] and catalytic properties [3,4]. These properties vary with size, shape and physicochemical properties of the Au NPs [5,6]. To integrate Au NPs in nanoelectronic devices, they need to meet requirements, such as precise positioning, good electrical contact, reliable anchoring to substrates and chemical stability, which remain challenges that are faced by nanodevice-related research. Therefore, the properties of Au NPs should be manipulated or tailored by introducing functional ligands on Au NPs, and the resulting assembly properties and connectivity to substrates and other nanostructures should be assessed.

For nanoelectronics applications, robust couplings of a defined number of ligands connecting nano building blocks is an important prerequisite to exhibit quantum effects due to the electron confinement of a nanostructure. Numerous top-down approaches have been developed to connect molecules or building blocks of interest, for example, via electro-deposition [7], break junction [8], ion-beam sputtering [9], electron migration [10] and so forth. By these top-down approaches, connecting molecules or nanoparticles to external leads is not trivially achieved. Irregular shapes of external leads and varying gap sizes are often observed. On the other hand, bottom-up approaches offer more flexibility to design nanostructures [11–13]. Via interfacial reactions between building blocks, nanostructures are fabricated. When performed in solution, the configuration of nanostructure complexities is hard to control. Yet, prominent studies have demonstrated well-controlled nanostructure configurations involving DNA-functionalized Au NPs [14–16]. Herein, the nanostructure assembly is governed by DNA hybridization, while the resulting configurations are determined by numbers of DNA molecules on Au NPs and are, therefore, self-limiting. However, to facilitate electron tunneling between nano building blocks, the length of ligand modifiers used should be limited to ≤2 nm. So far, common molecular modifiers used in nanoelectronic devices are dithiol-based ligands. Dithiol ligands are usually introduced after deposition of the NPs onto a substrate, because they cause severe aggregation of Au NPs when used in solution [17]. In this way, it is not trivial to control the number of ligands connecting the nanoparticles or building blocks.

Since the optical and electronic properties of Au NPs are size- and shape-dependent, the development of a robust coupling strategy should ideally be applied to a wide range of sizes. Citrate-stabilized Au NPs are most suited for nanoelectronic applications, as they are available in a wide range of sizes [18–20]. There are numerous procedures describing the synthesis of Au NPs and their functionalization strategies. However, many of those strategies can only be applied to monolayer-protected nanoclusters (MPCs), which are available only in a much narrower size range (<10 nm) [21–23]. Starting from amino-functionalized particles, larger MPCs, functionalized with thiols by ligand exchange, have also been used, and nanostructures have been made with other particles using azo and amido linkages [24].

Unlike MPCs, the colloidal stability of citrate-stabilized Au NPs in aqueous solution is mostly dependent on the surface charge, and thus, functionalization of citrate-stabilized Au NPs may compromise their colloidal stability: upon higher concentrations of the ligands introduced, the surface charge may be reduced drastically, which leads to irreversible precipitation of the functionalized Au NPs. To maintain the colloidal stability in aqueous solution, thiolates with carboxylic groups are commonly used either as the functional groups or as coadsorbents in ligand mixtures [25–27]. The functional groups can only be introduced on such Au NPs in a relatively low concentration in order to maintain colloidal stability. The choice of the thiol pairs in a ligand mixture should also be considered carefully (*i.e.*, ratio, chain length and so forth) to ensure equal affinity to the Au NPs. In solution, Au nanorods and citrate-stabilized and ligand-functionalized Ag NPs have been coupled using click chemistry [28].

The functionalization route also determines the reactivity of the functional groups on the Au NPs and, finally, the subsequent coupling yield. The reactivity of a functional ligand attached to a particle is affected by (i) the concentration of the ligand, which is determined by both the surface coverage of the ligand and the particle concentration in solution; (ii) the size of Au NPs used, which determines the diffusivity of the particles and, thus, the collision frequency of the functional ligand; and (iii) other, e.g., steric, factors influencing the inherent reactivity of the functional ligand upon attachment to a particle. With regard to the influence of NP size on reactivity, the nanoparticle mobility in solution decreases with increasing diameter. Larger nanoparticles are usually present in lower particle concentrations, and consequently, introducing low coverages of functional ligands on larger nanoparticles reduces the overall reactivity significantly.

**Scheme 1:**
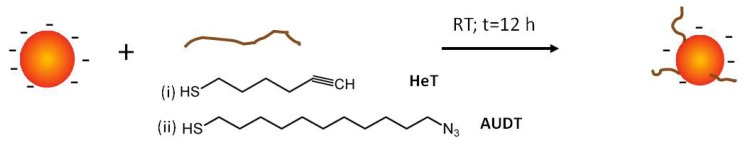
Functionalization of citrate-stabilized gold nanoparticles (Au NPs).

Here, we present an approach to connect functionalized nanoparticles to substrates via a few reliable, robust covalent couplings. The Huisgen 1,3-dipolar cycloaddition has been proven to yield stable molecular linkages prepared under mild conditions [29–31]. The presence of Cu(I) catalyst accelerates the reaction kinetics and enables one to perform the click reaction at ambient temperature. Thus, it provides a reliable chemical reaction to probe the stability and robustness of the resulting coupling bonds, and it allows investigation of the parameters influencing particle-bound ligand reactivity in a more systematic manner. The Huisgen click reaction has here been applied to probe the chemical reaction of functionalized nanoparticles to surfaces. Chain-like assemblies or aggregates of nanoparticles as a result of selective reaction have been reported [32–37]. We chose azido undecanethiol (AUDT) and hexynylthiol (HeT) as functional ligands for the click reaction (see Scheme 1). The concentrations of the ligands are varied, and the reactivity of the functional groups on the Au NPs is assessed, with an emphasis on the low surface coverage regime. Surface patterning by soft lithography is employed to allow a direct assessment of binding selectivity.

## 2. Results and Discussion

### 2.1. Functionalization of Au NPs

Citrate-stabilized Au NPs were functionalized with AUDT or HeT by following the procedure shown in Scheme 1. When performing the functionalization overnight, the incoming thiolates displaced citrate anions from the surface atoms due to their higher binding energy [38]. Noting that our aim is to functionalize Au NPs with a low coverage of ligands, different sets of thiolate concentrations were introduced, depending on the fraction of surface atoms occupied. For a starting colloidal solution concentration of 2.85 × 10^12^ NPs mL^−1^, whereby each 10 nm diameter Au NP has approx. 4,400 surface atoms, gives a total concentration of surface atoms of 21 μM. For such large Au NPs, the maximum coverage of alkanethiol ligands is 33%, which is similar to that of flat Au(111) [39,40]. The percentages given below correspond to the fractions of added thiol relative to the concentration of surface Au atoms (so full coverage would correspond to 33%).

As Au NPs are optically active, a change in the colloidal stability upon functionalization can be detected by UV-Vis spectroscopy. Position, intensity and width of the absorption band are strongly dependent on the size and shape of the Au NPs. For gold nanoparticles with a diameter of 10 nm, the surface plasmon resonance band in aqueous solution is found at 525 nm. No difference was observed in the peak position and shape of the absorbance spectra of the starting solutions. This demonstrates that the colloidal stability is maintained. In contrast, when the colloidal stability is disturbed or Au NPs undergo aggregation, their interparticle distances become smaller than the diameter of a single Au NP, which results in peak broadening and/or peak shifting at a higher wavelength.

**Figure 1:**
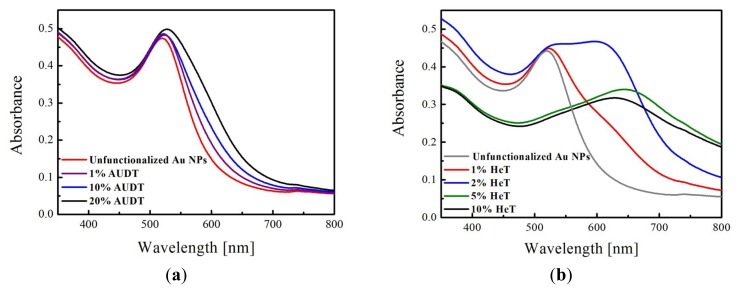
Absorbance spectra of Au NPs (*d* = 10 nm) before and after place-exchange reaction with azido undecanethiol (AUDT) (**a**) and hexynyl thiol (HeT) (**b**).

As shown in Figure 1, the absorbance spectra, depending on percentages of AUDT or HeT concentrations on the Au NPs, were recorded. We observed different behavior of peak shifting, as well as peak broadening, depending on the thiol introduced (AUDT or HeT) and the corresponding concentrations. For example, adding 0.21 μM of AUDT to citrate-stabilized Au NPs (which is equal to 1% the amount of surface atoms), the absorbance peak is shifted only slightly, from 525 nm to 527 nm. Noticeable peak shifting to 540 nm and peak broadening at around 600 nm were seen as the AUDT concentration was increased to 10% and 20%, which indicate fractions of Au NPs form aggregates. On the other hand, a much more drastic shifting and broadening of absorbance peaks were recorded as the citrate-stabilized Au NPs were functionalized by HeT with similar concentration ranges. For example, for 1% HeT, the peak absorbance was shifted to 530 nm, but more interestingly, the shoulder is broadened at 630 nm, indicating aggregation of the functionalized Au NPs due to reduced colloidal stability. A large broad peak was even developed as the HeT concentration is doubled, and the peak at 530 nm diminishes as higher concentrations of HeT are introduced (5% and 10%).

Dynamic light scattering (DLS) provides additional analysis of the aggregation behavior of the functionalized Au NPs by providing the hydrodynamic diameter (*D*_h_) of nanoparticles or aggregates. Herein, we investigated the aggregation behavior of these functionalized Au NPs and correlate them to the UV-Vis spectra above (Figure 1). The results are summarized in Table 1. In the low concentration regime (*i.e.*, 1% and 2%), *D*_h_ of Au NPs functionalized with AUDT changed only slightly compared to unfunctionalized Au NPs (*D*_h_ = 10 ± 1 nm). Aggregation was observed at higher concentrations, such as at 10%. On the other hand, in the case of HeT, significant changes in *D*_h_ were already recorded at low concentrations. For 1% HeT, the absorbance peak broadening and development of a new absorbance peak at 630 nm seems to give an effect to the overall *D*_h_, even though a pronounced peak is still observed at 530 nm (Figure 1b). Au NPs with 2% HeT suffer from aggregation, yet not precipitation. At higher concentrations, the Au NPs precipitate and are difficult to be measured by DLS.

Since the ligands are introduced in at low coverages, it is of importance that those functional groups are well-exposed. Recently, Wang *et al*. described an intermediate phase of ligand-exchange, whereby island-like domains containing functional ligands were observed [41]. Interestingly, no lying-down configuration of functional ligands was observed, which may impact the reactivity of the ligands at subsequent interfacial reaction. Thus, it is of our interest to probe the reactivity of the low-coverage functionalized Au NPs.

### 2.2. Covalent Bonding of Functionalized Au NPs onto Surfaces

**Figure 2:**
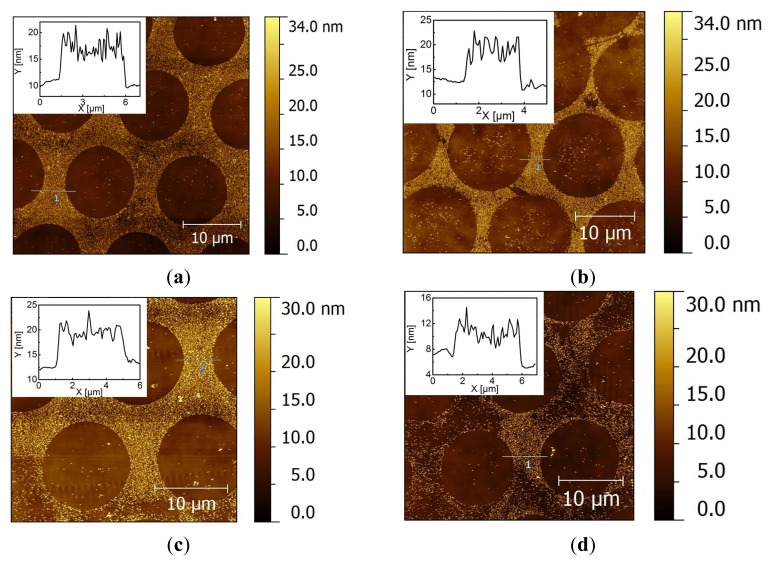
Tapping-mode atomic force microscopy (AFM) images after deposition of unfunctionalized citrate-stabilized Au NPs on Au samples microcontact printed with mercaptohexadecanoic acid (MHDA) and backfilled with HeT (**a**, **b**) and AUDT (**c**, **d**), after washing in PBS (**a**, **c**) and subsequent sonication in PBS for 10 min (**b**, **d**).

**Scheme 2:**
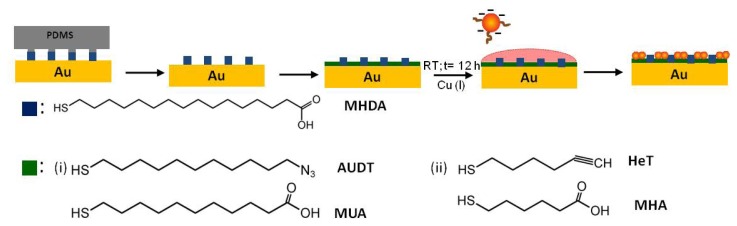
Substrate preparation and selective reactivity of functionalized Au NPs.

The procedure to perform the reaction of functionalized Au NPs on a surface is shown in Scheme 2. For our purpose, it is crucial to distinguish between physical (*i.e.*, van der Waals) and chemical (*i.e.*, covalent) driving forces of the immobilization of Au NPs onto substrates. To study potential physical driving forces, we used unfunctionalized citrate-stabilized Au NPs. We first investigated the selective deposition of citrate-stabilized Au NPs on a patterned monolayer. A freshly prepared polydimethylsiloxane (PDMS) stamp with protruding round dots of 10 μm was inked with a 2 mM ethanolic solution of mercaptohexadecanoic acid (MHDA) and backfilled with a monolayer of pure HeT or AUDT. After Au NP immobilization and washing treatments, the substrates were characterized by atomic force microscopy (AFM) in tapping mode (Figure 2).

AFM images show that citrate-stabilized Au NPs preferably deposit on the backfilled areas containing either HeT or AUDT (Figure 2). In addition, the height profiles show that single layers of Au NPs are deposited on the backfilled areas. It indicates that the colloidal stability of Au NPs is maintained during assembly and that the assembly is self-limiting. The nature of the negatively charged citrate anions decorating the Au NPs results in an electrostatic repulsive force in the stamped MHDA areas [42]. In contrast, citrate-stabilized Au NPs are attracted to the backfilled areas by Van der Waals forces [43,44]. The preference of the Au NPs for the backfilled areas is problematic, because we expect to have a covalent coupling reaction in those backfilled areas (*i.e.*, on HeT or on AUDT) when using functionalized NPs. Washing the samples in a stream of PBS did not remove the Au NPs from the backfilled areas. Sonication, which is more powerful, can potentially be used to overcome the attractive forces. Yet, even after sonication in PBS (pH 9) for 10 min, AFM images showed that most NPs remained on the HeT-backfilled areas (Figure 2b). At the same time, some Au NPs were detached from the AUDT areas (Figure 2d). This result implies that the negative charge of the azide functional groups may play a role in weakening the attractive forces. We also found that 10 min is the maximum time in which the gold layer can withstand the treatment.

**Figure 3:**
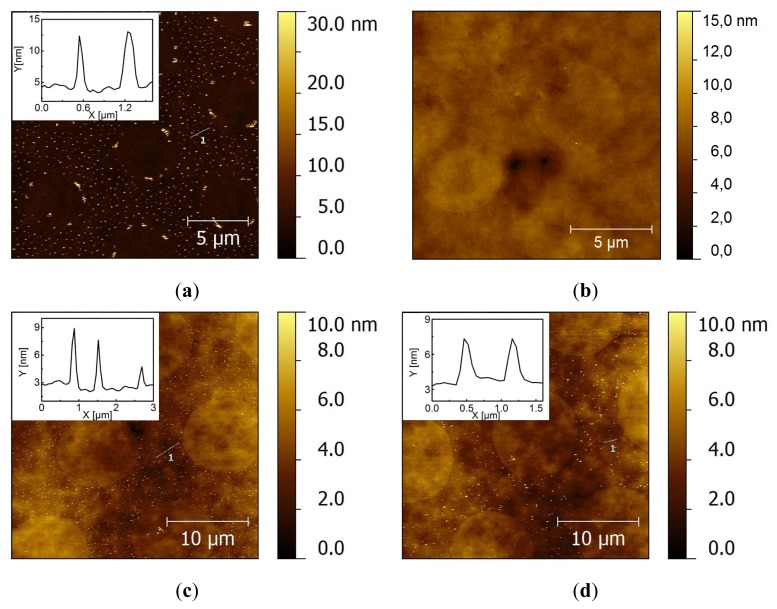
Tapping-mode AFM images after deposition of citrate-stabilized Au NPs onto backfilled mixed monolayers: AUDT/mercaptoundecanoic acid (MUA) (**a**, **b**) and HeT/mercaptohexanoic acid (MHA) (**c**, **d**) to suppress nonspecific adsorption, after washing in a stream of PBS (**a**, **c**) and sonication in PBS for 10 min (**b**, **d**).

To suppress these nonspecific interactions, we mixed the functional molecules present on the substrate with a coadsorbent to repel the charged NPs. Mercaptohexanoic acid (MHA) and mercaptoundecanoic acid (MUA) were chosen as co-adsorbents, because they have the same chain length as HeT and AUDT, respectively, so that they can be expected to provide statistically mixed SAMs. Thus, after stamping MHDA, the substrates were immersed in a mixed thiol solution containing equimolar amounts of functional thiol and its coadsorbent. From AFM images, we observed much less nonspecific adsorption on the backfilled areas after washing the substrate with PBS (pH 9), as expected (Figure 3a,c). After subsequent sonication for 10 min, the mixed backfilled areas containing MHA and HeT did not show any significant detachment of Au NPs (Figure 3d). On the other hand, backfilled areas containing AUDT and MUA showed that most of the physisorbed Au NPs were washed away (Figure 3b). Alternatively, we also used mercaptoundecanol (MUD) as the co-adsorbent, which yields hydrophilic, neutral SAMs. After washing treatments, we observed severe physisorption of Au NPs, persisting even after 15 min of sonication (Figure S1). All-in-all, these results show that the nonspecific Van der Waals interactions can be suppressed by providing sufficient electrostatic repulsion, as has been also widely demonstrated in the adsorption of proteins on monolayers. However, depending on the properties (e.g., polarity) of the functional groups, HeT provides a stronger attractive force than AUDT, as also observed in Figure 1.

As the next step, we probed the reactivity of Au NPs functionalized by a low coverage of ligands. Considering Au NPs with a diameter of 10 nm, the maximum coverage (33%) is similar to a flat Au(111) surface, which is equal to 1,452 surface atoms with an area per molecule of 0.216 nm^2^. The ligands expected to contribute to the click reaction are only those located at the contact area. Considering that the shape of a Au NP is spherical, the contact area is equal to a hemispherical cap area (*A*_c_, see Figure S2). Taking into account the hemispherical area of surface atoms with cap height, *h*_c_, is similar to the diameter of a gold atom (0.28 nm), *A*_c_ gives 8.5 nm^2^. Assuming that the ligands are distributed evenly on the Au NPs, *i.e.*, either on AuNP/1% HeT or on Au NP/1% AUDT, there are in total 44 functional ligands covering each Au NP. Thus, the average number of ligands located at the contact area is 1.2. This indicates that we expect a particle to react with only a single covalent bond to the surface. This estimation is close enough for the ultimate requirement of single electron–based nanoelectronic devices.

**Figure 4:**
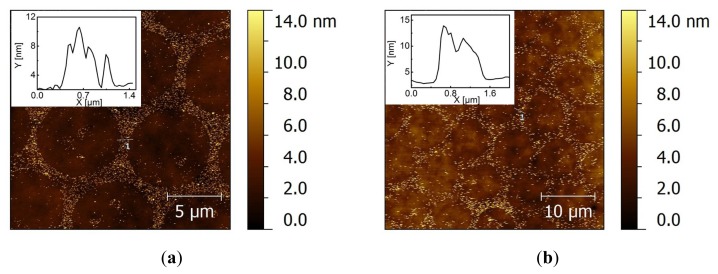
Tapping-mode AFM images after coupling of Au NP/1%HeT onto backfilled AUDT/MUA areas as a result of Cu(I)-catalyzed click reaction, after washing in a PBS stream (**a**) and after sonication in PBS for 10 min (**b**).

We found that there is still substantial nonspecific adsorption of Au NPs on HeT/MHA backfilled monolayers, as shown above (Figure 3c,d). This gives a rise to difficulties in comparing the adsorption yields of the covalent coupling of Au NP/1% AUDT. On the other hand, physisorbed citrate-stabilized Au NPs are completely desorbed from AUDT/MUA mixed monolayers (Figure 3a,b). Therefore, we chose to investigate the reactivity of Au NP/1% HeT on AUDT/MUA mixed monolayers following the procedure depicted in Scheme 2. Even though *D*_h_ recorded by DLS shows that Au NP/1% HeT suffers from aggregation, there is a pronounced absorbance peak present at 530 nm, which indicates that part of Au NP/1% HeT maintains its colloidal stability in solution. The substrate was prepared in a similar way as previous experiments, except a Cu catalyst was introduced to the colloidal solution to allow the Cu(I)-catalyzed click reaction. The substrates were subsequently washed in a PBS stream and sonication in PBS (pH 9) for 10 min. After each washing treatment, AFM images were taken (Figure 4). We observed a significant preference of Au NP/1% HeT to the backfilled area. More importantly, a significant number of Au NPs can withstand the sonication treatment. The density of deposited Au NPs was estimated using gwyddion [45] and gave an average density of 23 ± 6 NP μm^−2^. Some particles (2 ± 1 NP μm^−2^) were nonspecifically deposited on the stamped MHDA (dots) areas, which is probably due to some local exchange reaction during backfilling. After sonication for 10 min., many nanoparticles were still significantly present on the backfilled area, with a particle density of 6 ± 3 NP μm^−2^. In addition, the height profiles do not indicate any clustering of Au NPs bound onto the substrate, which implies that 1% of functional ligands is sufficient to provide covalent coupling of the NPs to a substrate, probably through single covalent bonds. Interestingly, when the ratio of AUDT: MUA in the backfilled areas was increased to 1:3, the selective covalent coupling of Au NP/1%HeT on the substrate was still observed, whereas unfunctionalized or citrate Au NPs were washed away (Figure S3). The results also suggest that only NPs with well-exposed HeT groups are reactive towards surface-bound AUDT, whereas the aggregates and potentially unfunctionalized Au NPs are washed away. We tried to accelerate the assembly kinetics by performing the click reaction at elevated temperature (60 °C). However, no specific nanoparticle assembly was observed (Figure S4), and the nanoparticles tended to be aggregated. We also observed a colloidal instability of the nanoparticle dispersion at this temperature, which probably prevents proper binding to the substrates.

## 3. Experimental Section

All reagents and solvents, including MHDA, MUA, MHA, MUD, CuSO_4_ 5H_2_O, sodium ascorbate and ethanol, were used as received and were purchased from Sigma–Aldrich, unless otherwise noted. Au NPs (*d* = 10 ± 1 nm) were purchased from BBInternational (Cardiff, UK). HeT and AUDT were purchased from Prochimia (Sopot, Poland). Polydimethylsiloxane (PDMS; Sylgard 184) was purchased from DowChemical. Milli-Q water with a resistivity greater than 18 MΩ cm was used in all experiments.

Prior to functionalization, so-called starting solutions were prepared by mixing stock solutions of NPs (as purchased) with ethanol in an equivolume ratio. Functionalization of the Au NPs with either ligand was performed by addition of different volumes of a ligand stock solution providing the desired coverage (here, expressed in percent relative to the number of Au surface atoms, assuming that a Au NP of 10 nm in diameter has 4400 surface atoms). The ligand concentrations are thus varied from 1%, to 20%. For example, adding a ligand to the NP solution yielding a ligand concentration of 0.21 μM provides an average coverage of 44 thiolates per nanoparticle (equal to 1% of the amount of surface atoms). The ligand exchange was carried out overnight at room temperature. Since only a small fraction of surface atoms are functionalized, complete ligand attachment is assumed and no washing steps were conducted. The colloidal stability of functionalized Au NPs and the corresponding *D*_h_ were measured by UV-Vis Spectrometer (Perkin Elmer lambda 850, Perkin Elmer, Waltham, MA, USA) and DLS (Malvern Instruments Ltd., Malvern, UK), respectively. The measurements were repeated three times at room temperature.

Gold substrates were prepared by the template stripping technique [46]. Stamps for microcontact printing (μCP) were molded on a dot-featured silicon master (*d* = 5 and 10 μm) using Sylgard 184 poly(dimethylsiloxane) prepolymer and curing agent (10:1 *v*/*v*). The curing step was done at 60 °C overnight. The stamps were cut into pieces of 1 × 1 cm^2^. Prior to stamping, a freshly prepared stamp was inked with 2 mM MHDA in ethanol for 5 min. Subsequently, the stamp was blown dry under nitrogen flow and directly brought into contact to a freshly template-stripped gold substrate. Replicated dot features on PDMS containing MHDA were then transferred to the gold substrate for 2 min. The substrate was subsequently immersed into a solution of a single thiol (*i.e.*, AUDT or HeT) or a mixture of thiols (AUDT or HeT with as its co-adsorbent, MUA or MHA, respectively) of 3 mM total concentration for 3 h. The substrate was then washed with ethanol and blown dry in a nitrogen stream. The subsequent nanoparticle assembly was carried out by immersing the patterned substrate into the starting solution of the native citrate-stabilized or functionalized gold nanoparticles solution. For performing the click reaction, 20 μM of Cu(II)SO_4_ 5H_2_O and 1 mM sodium ascorbate was added to the colloidal solution of functionalized nanoparticles. After overnight immersion, the substrates were washed in phosphate buffer (pH 9), followed by sonication in phosphate buffer (pH 9) for 10 min. After each washing step, the substrates were characterized by AFM (Digital Instruments DI3100a) in tapping mode. A silicon nitride tip was used with a force constant of 10–130 N/m. All measurements were performed in air at room temperature.

## 4. Conclusions

Patterned SAMs have been used to investigate the reactivity of Au NPs functionalized with a low coverage of functional ligands via catalyzed click reaction in aqueous solution. Prior to the coupling reaction, functional groups were introduced on the Au NPs while maintaining the colloidal stability. Washing treatments were developed to allow reliable determination of the reactivity of the coupling reaction. The results revealed that an average number of 44 ligands on the 10-nm Au NPs, whereby only about one or two ligands are present in the contact area with a substrate is sufficient to chemically couple the Au NPs to a surface. The method may allow careful design of complex systems with control over the number of bonds in coupling nanoparticles or nano building blocks.

## Figures and Tables

**Table 1:** Hydrodynamic diameter (*D*_h_) of functionalized Au NPs.

Concentration (%)	1	2	10
*D*_h_ (AUDT) (nm)	14 ± 3	14 ± 3	57 ± 4
*D*_h_ (HeT) (nm)	84 ± 3	81 ± 3	precipitates

## Supplementary Files

Supplementary File 1 Supplementary Information (PDF, 2058 KB)
