# Supplementary material for: Covalent Coupling of Nanoparticles with Low-Density Functional Ligands to Surfaces via Click Chemistry
Source: Int J Mol Sci. 2013 Feb 7;14(2):3705–17. doi: 10.3390/ijms14023705 (PMC3588066; doi:10.3390/ijms14023705)

Supplementary Information

1. Estimation of Surface Atoms per Particle and Surface Functionalization

The amout of surface atoms is estimated from the total of atoms depending on its diameter. The number of surface atoms can be estimated by following equation:

$$N_{T-\mathrm{Au}} = \frac{\frac{4\pi}{3}\left(\frac{d}{2}\right)^3}{v_{\mathrm{Au}}} \tag{1}$$

where d is diameter of a nanoparticle, $v_{au} \approx 1.7 \times 10^{-2}$ nm^3.

2. Physisorption of Nanoparticles on Backfilled Mixed Monopayer

Figure S1. AFM micrographs of citrate-stabilized nanoparticles deposited on backfilled AUDT-MUD equimolar mixed monolayers. (**a**) After washing under stream of PBS (pH 9) and (**b**) after sonication in (pH 9) 15 min.

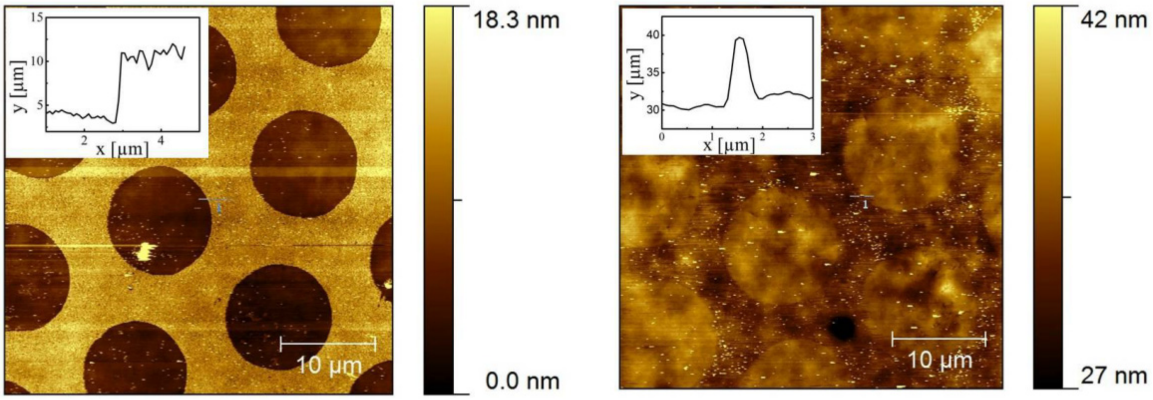

(**a**) (**b**)

3. Hemisperical Cap Area of a Sphere

Figure S2. Schematic of spherical Au NPs.

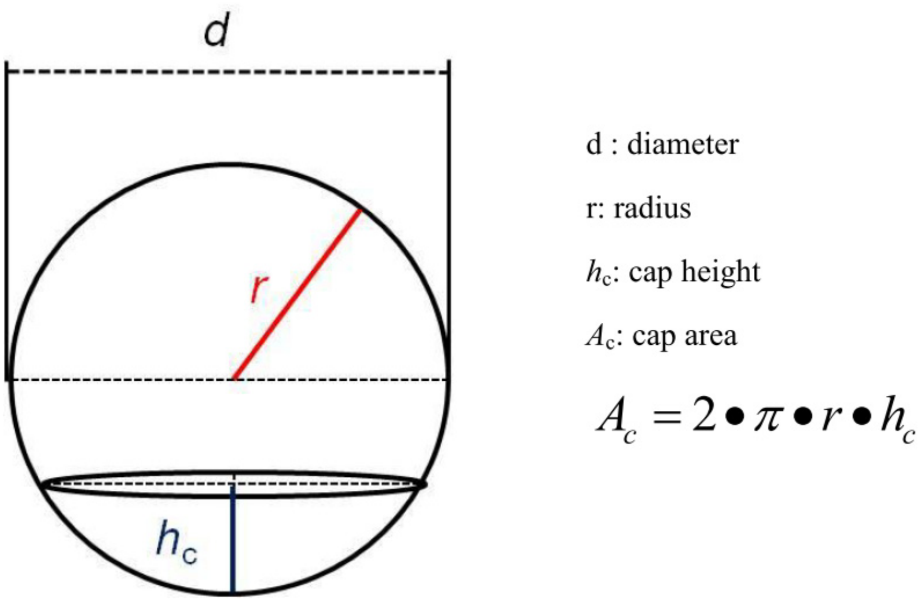

4. Click Reaction of Au NP/1% HeT at Low AUDT Ratio on Substrates

Figure S3. Topographic AFM micrographs of assembled AuNP/1 HeT (**a**,**b**) and AuNP/citrate (**c**,**d**) on Au/(MUA:AUDT = 3:1) via click reaction. (**a**,**c**) after washing under stream of PBS and (**b**,**d**) after sonication for 10 min.

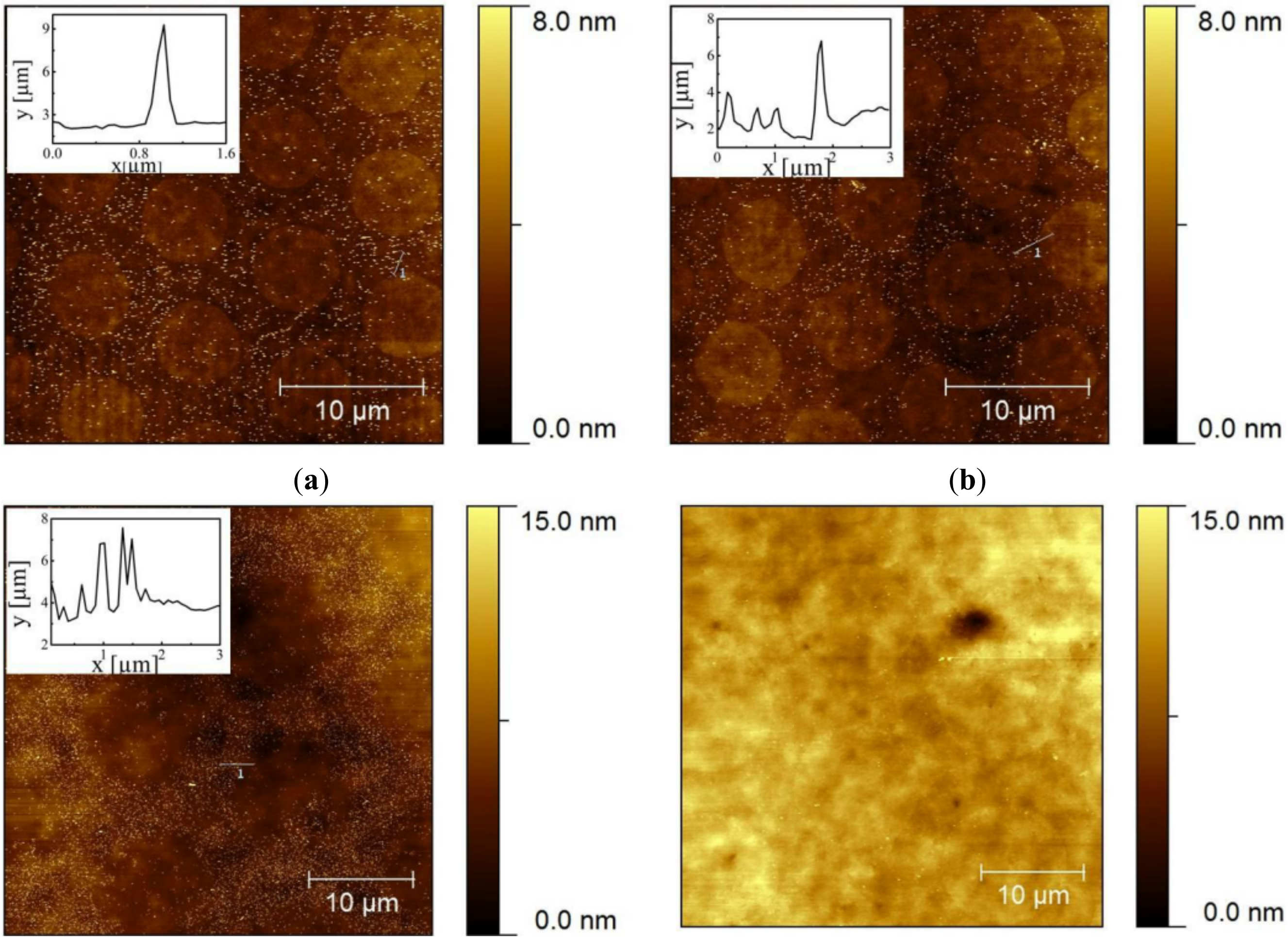

(**a**) (**b**) (**c**) (**d**)

5. Click Reaction at 60 °C

Figure S3. Topographic AFM micrographs of assembled AuNP/1 HeT on Au/(MUA:AUDT = 1:1) via click reaction at 60 °C.

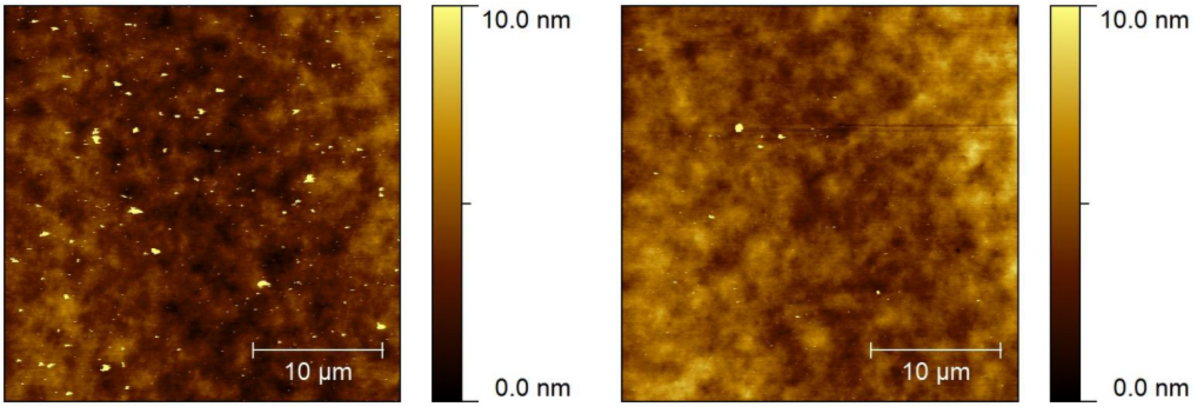

Supplement: Supplementary File 1 — Supplementary Information (PDF, 2058 KB) [file ijms-14-03705-s001.pdf]
